# Tea Drunkards

**Published:** 1874-04

**Authors:** 


### TEA DRUNKARDS.

Dr. Arlidge, one of the Pottery Inspectors of Staffordshire, has put forth a very sensible protest against a very pernicious custom which rarely receives sufficient attention either from the medical profession or the general public. He says that the women of the working classes make tea a principal article of diet, instead of an occasional beverage. They drink it several times a day, and the result is a lamentable amount of sickness. This is no doubt the case, and, as Dr Arlidge remarks, a portion of the reforming zeal directed against intoxication might be wisely diverted to the repression of this very serious evil of tea-tippling among the lower classes. Tea, in anything beyond moderate quantities, is as distinctly a narcotic poison as is opium or alcohol. It is capable of ruining the digestion, of enfeebling and disordering the heart's action, and of generally shattering the nerves. And it must be remembered that not merely is it a question of narcotic excess, but the enormous amount of hot water which tea-bibbers necessarily drink, is exceedingly prejudicial both to digestion and. nutrition. In short, without pretending to place this evil on a par as to general effect with those caused by alcoholic drinks, one may well insist that our teetotal reformers have overlooked, and even to no small extent encouraged, a form of animal indulgence which is as distinctly sensual, extravagant and pernicious as any beer-drinking or gin swilling in the world.—*Boston Medical and Surgical Journal.*

